# Macrophage Plasticity and the Role of Inflammation in Skeletal Muscle Repair

**DOI:** 10.1155/2013/491497

**Published:** 2013-01-30

**Authors:** Yacine Kharraz, Joana Guerra, Christopher J. Mann, Antonio L. Serrano, Pura Muñoz-Cánoves

**Affiliations:** ^1^Cell Biology Group, Department of Experimental and Health Sciences, Pompeu Fabra University (UPF) and CIBER on Neurodegenerative Diseases (CIBERNED), 08003 Barcelona, Spain; ^2^Institució Catalana de Recerca i Estudis Avançats (ICREA), 08003 Barcelona, Spain

## Abstract

Effective repair of damaged tissues and organs requires the coordinated action of several cell types, including infiltrating inflammatory cells and resident cells. Recent findings have uncovered a central role for macrophages in the repair of skeletal muscle after acute damage. If damage persists, as in skeletal muscle pathologies such as Duchenne muscular dystrophy (DMD), macrophage infiltration perpetuates and leads to progressive fibrosis, thus exacerbating disease severity. Here we discuss how dynamic changes in macrophage populations and activation states in the damaged muscle tissue contribute to its efficient regeneration. We describe how ordered changes in macrophage polarization, from M1 to M2 subtypes, can differently affect muscle stem cell (satellite cell) functions. Finally, we also highlight some of the new mechanisms underlying macrophage plasticity and briefly discuss the emerging implications of lymphocytes and other inflammatory cell types in normal versus pathological muscle repair.

## 1. Introduction

Tissue regeneration is an evolutionary conserved process in which interactions between infiltrating inflammatory cells and resident cells must be finely coordinated if homeostasis and functionality are to be restored. Perturbation of these interactions leads to unsuccessful regeneration and often compromises survival of the individual [1, 2]. Skeletal muscle, the most abundant tissue of the body, is essential for breathing, posture maintenance, and locomotion, besides serving important homeostatic and metabolic roles, such as heat production and carbohydrate or amino acid storage. Loss of muscle functionality in acute or chronic conditions results in diminished mobility and strength, in addition to metabolic disorders, which can have potentially lethal consequences. Abnormal muscle repair can occur in the context of persistent myofiber degeneration and/or inflammatory infiltration, such as in Duchenne muscular dystrophy (DMD), or when extracellular matrix (ECM) deposition is excessive or inappropriately timed, eventually leading to the substitution of the normal muscle architecture by fibrotic tissue [3]. Therefore, preservation of the capacity of skeletal muscle to regenerate in a coordinated manner in response to direct mechanical trauma (acute injury), or following secondary damage as a consequence of genetic neuromuscular alterations, is of utmost importance. 

## 2. Injury-Induced Skeletal Muscle Regeneration: A Model for Tissue Repair

**Figure 1:**
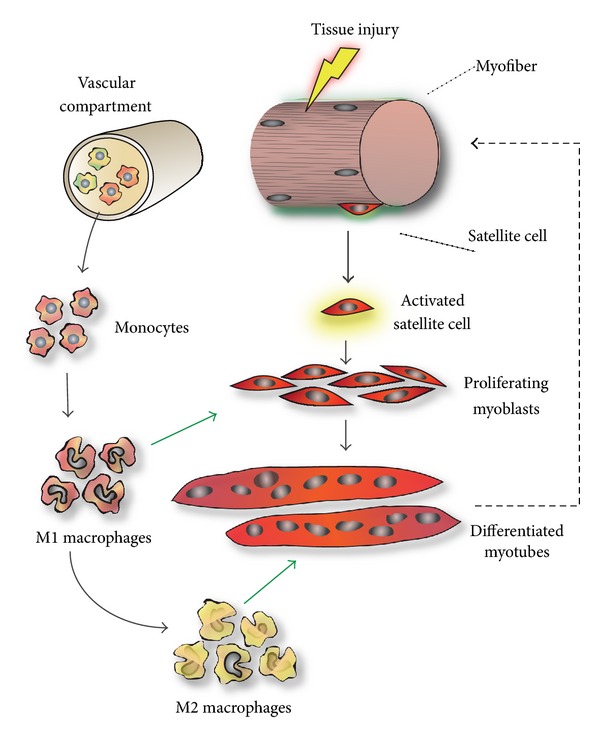
Inflammation and macrophage polarization in skeletal muscle injury and repair. Satellite cells are muscle-resident stem cells which are located underneath the basal lamina of myofibers and are normally quiescent (top right). Upon muscle injury, satellite cells get activated, start to proliferate as myoblasts, and subsequently fuse and differentiate into myotubes that later grow thereby replacing damaged muscle. Several cell types influence the outcome of regeneration, in particular inflammatory cells released from the blood (top left). Proinflammatory monocytes and neutrophils (not shown) extravasate shortly after damage, invading the injured areas where they differentiate into proinflammatory macrophages that phenotypically resemble M1 macrophages. These cells clear the damage and release a number of cytokines that stimulate myoblast proliferation. M2-like macrophages are present locally at later stages of regeneration acting as promoters of myoblast differentiation and fusion. Other cell types such as mast cells and lymphocytes also have less defined roles in muscle repair (not shown).

The capacity of muscle to regenerate relies primarily on a specific population of normally quiescent muscle stem cells, named satellite cells due to their particular position and intimate association with muscle fibers [4]. Many additional cell types also play a role in efficient tissue repair, including resident cells within the skeletal muscle niche such as PICs (PW1^+^ interstitial cells), mesoangioblasts, FAPs (fibro/adipogenic progenitors), and other ECM-associated cells [5]. However, the inflammatory cells that infiltrate the injured muscle appear to be the most critical, alongside satellite cells, for successful regeneration. Among these inflammatory cells, it is the monocytes/macrophages which play the greatest role in this repair process (Figure 1). In response to local vascular damage and signals released by degenerating myofibers, these cells extravasate from the blood and infiltrate the injured areas, to phagocytose myofiber debris. In addition to this critical function, inflammatory cells produce growth factors, cytokines, inflammatory mediators, and damage signals that have a profound impact on satellite cell behavior during the repair process [6]. In concert with monocyte/macrophage recruitment, quiescent satellite cells are activated by damage/inflammation-associated signals and begin to proliferate, thereby providing a sufficient supply of myonuclei for the formation of new myofibers. While most of the proliferating satellite cells will commit to myogenic differentiation, a small population will undergo self-renewal and replenish the pool of quiescent satellite cells, thus maintaining muscle stem cell homeostasis [7]. 

A further critical step in the repair process is the re-establishment of the ECM around the individual fibers and bundles which helps strengthen the muscle and provides additional support for contraction. Correct remodeling and reorganizing of the muscle ECM after damage is necessary for providing new scaffold structures over which nascent myofibers will be formed, as well as ensuring correct spatial organization of the new myofibers [8]. Excessive and persistent ECM deposition (fibrosis) leads to failure in restoring the previous structure of myofibers, thus provoking a defective regenerative outcome. Although several studies have shown that satellite cell-derived myoblasts may synthesize many components of the ECM, the major matrix-producing cell is the fibroblast [9]. Like satellite cells, resident fibroblasts proliferate and migrate to the injury site immediately after muscle damage, where they function in close proximity to satellite cells and regenerating myofibers. Indeed, recent findings have demonstrated the relevance of the interplay between satellite cells and fibroblasts and/or FAPs as a determinant factor for the efficiency of the repair process [10–12]. Specific deletion of fibroblasts using genetic approaches resulted in impaired regeneration due to the lack of proliferation of satellite cells and their premature differentiation, strongly suggesting a paracrine action of fibroblasts on muscle cells [10]. An important part of the functional role of ECM in controlling the process of repair is carried out by the basal lamina, a thin layer of nonfibrillar collagen, noncollagenous glycoproteins, and proteoglycans that is in direct contact with the myofiber plasma membrane (see [13] for review). The basal lamina also surrounds satellite cells forming part of the niche that is necessary for maintaining the stem-like properties of quiescent satellite cells. Because of this direct satellite cell contact, the basal lamina composition and integrity also influence the process of repair, by providing guidance cues for satellite cell migration. In the normal repair process, prevention of excessive accumulation of ECM components and restoration of the original basal lamina integrity are controlled by the balanced activities of extracellular proteases and their inhibitors. Dysregulation of these enzymatic activities may cause unrestricted ECM accumulation and altered basal lamina composition, which eventually could lead to fibrosis development and loss of normal muscle architecture [14]. Lastly, proangiogenic factors also need to operate at advanced stages of the repair process to revascularize the newly formed myofibers, thus restoring the vascular network of the damaged tissue [15]. For a recent and comprehensive review focused on macrophage biology in skeletal muscle injury, muscle disease, and fibrosis, see Bosurgi et al. (2011) [16]. In this paper, we focus more specifically on the current knowledge of the inflammatory control of satellite cell-dependent muscle repair in acute injury and highlight several recent findings. 

## 3. Inflammation in Efficient Muscle Repair 

Just as satellite cells go through a controlled process of activation from quiescence, proliferation, and self-renewal, and finally differentiation and fusion into new myotubes, the inflammatory response also undergoes a series of carefully regulated stages to ensure an efficient return to tissue homeostasis. That is, the composition of the inflammatory infiltrate is dynamically regulated to facilitate timely initiation of divergent functions, while the duration and intensity of the various inflammatory components must also be coordinated with the degree of muscle damage and the need to change tissue milieu during repair [3, 6, 17]. For example, interfering with the inflammatory response immediately after acute injury disrupts the phagocytosis of necrotic fibers and impedes seeding of new myofibers. Just as detrimental is the prolongation of inflammation which can promote muscle degeneration and fibrosis development, as occurs in severe myopathies such as DMD which are characterized by chronic inflammation [18]. Macrophages have recently been shown to promote survival and proliferation of myogenic precursor cells that were introduced into *mdx* skeletal muscle [19]. Thus, a tightly regulated, transient inflammatory response is required for normal muscle regeneration. Improving our understanding of the different cell subtypes and identifying the factors that regulate their function and the timing of their activity will enable us to improve pharmacological treatment of acute injury and neuromuscular disorders associated with chronic inflammatory responses.

## 4. Phases of the Inflammatory Response in Acute Muscle Injury

Most studies of skeletal muscle regeneration use acute models of injury and repair, such as sterile destruction of myofibers by either injection of toxins, such as cardiotoxin, notexin, or barium chloride, or by performing freeze crush injuries. These models are useful for synchronizing the repair processes and performing systematic studies, although they do not necessarily reflect the more physiologic repair associated with contraction injuries or replicate the different kinetics of chronic inflammation observed in myopathies. Moreover, there are important contributions of mouse strain to the inflammatory component and kinetics that are briefly discussed below and elsewhere [20, 21]. However, despite these variables, the inflammatory response to experimentally induced muscle repair follows an ordered pattern.

An immediate response to sterile muscle injury is the local activation of the innate immune response via the release of largely unknown factors, but which could include heat shock proteins, high mobility group box 1 (Hmgb1) as well as endogenous myofiber proteins and nucleic acids that become decompartmentalized as the fiber breaks and act as damage-associated molecular patterns (DAMPs) [22]. One of the earliest subsequent events is the invasion of the damaged site by inflammatory cells, particularly monocytes and polymorphonuclear leukocytes, which include neutrophils, that secrete proinflammatory cytokines and phagocytose particles (such as cellular or bacterial debris) [23]. Neutrophils constitute the first wave of inflammatory cells to enter the damaged tissue, reaching elevated numbers as soon as 2 hours after the initial injury. Neutrophils are, however, short-lived cells, whose number declines rapidly, probably through apoptosis, and they are essentially undetectable 3-4 days after injury [6]. The exact role of neutrophils in toxin-induced or freeze-crush injury is not clearly defined. However, several studies on contraction-induced injury show that neutrophils play a key role in repair by causing secondary damage, through the release of reactive oxygen species (ROS) and proteases, as well as facilitating phagocytosis and recruitment of monocytes by the release of cytokines [24, 25]. Neutrophils are known to enter into contraction-damaged muscle via a process called diapedesis that requires CD18 (integrin-*β*2) [25]. Interestingly, in contraction-injured CD18-deficient mice, neutrophil, but not macrophage, recruitment was impaired, while physiological signs of repair such as fiber size and force were more quickly restored compared to wild-type mice [25].

Recent studies have shown that resident macrophages in the muscle epimysium/perimysium connective tissue orchestrate the innate immune response to injury, which is linked to adaptive immunity through inflammatory dendritic cells (DCs) [26]. In addition to resident macrophages, blood monocytes also enter the damaged tissue and start differentiating into macrophages shortly after invasion by neutrophils [17]. Other inflammatory cell types, such as mast cells and T cells, have also been implicated in the repair and fibrogenesis of several tissues/organs; however, their role in muscle repair and/or fibrosis is generally limited (see also below) [27]. Monocytes originate in the bone marrow and circulate to the blood and the spleen before entering the muscle after injury [28]. They are equipped with chemokine and adhesion receptors that allow them to migrate from the blood to the injured tissues, where they produce proinflammatory cytokines and phagocytose dying or apoptotic cells. In the blood, circulating monocytes can be classified into at least two populations that are distinguishable by their expression levels of Ly-6C (also known as GR1) and of chemokine receptors CCR2 and CX3CR1 [29]. These two monocyte populations use different mechanisms for extravasation and probably have different functions. The GR1^+^ monocyte cell pool has been designated as the “inflammatory” population because they efficiently produce proinflammatory cytokines [30]. Through the CCR2/CCl2 axis, they are rapidly recruited to, and accumulate at, the site of inflammation [31, 32]. On the other hand, the GR1^−^ population of monocytes has an “anti-inflammatory” function, which includes supporting tissue repair and patrolling the vasculature [33]. In contrast to GR1^+^ cells, GR1^−^ monocytes enter damaged tissues in a CX3CR1-dependent manner just after the onset of inflammation in models of sterile injury [34–36]. An important consideration beyond the scope of this paper is the known heterogeneity in the use of cell surface markers between mice and humans, with human monocytes broadly defined as expressing different levels of CD14 and CD16 [37]. Thus, as most studies are performed in mice, care will be needed in trying to extrapolate findings to humans and the clinic.

Classically, there are believed to be two waves of tissue-infiltrating monocytes in most experimental wound healing models: a first wave comprising the GR1^+^ population, endowed with proinflammatory function and a second wave of GR1^−^ monocytes with an anti-inflammatory function. Interestingly, using an acute muscle injury model, Arnold and colleagues showed that the GR1^+^ monocyte population is the only one recruited to the injury site, switching subsequently within the damaged tissue into an “anti-inflammatory” macrophage population, thereby dampening the earlier proinflammatory wave and also supporting myogenesis [38]. Distinct macrophage populations have also been associated with the increased fibrosis observed in dystrophic muscle (see also below) [39]. Together these observations suggest that the mechanisms of leukocyte recruitment and maturation could be specific for each type of damage, tissue, and microenvironment.

## 5. Classification of Macrophage Populations in Tissue Repair: Specific Markers versus Functional Properties

As suggested by the experiments above and additional data from other tissue repair systems [40–42], macrophages exist as different functional populations at different times after injury. Generally, these populations are considered to exhibit opposing activities, being either polarized towards proinflammatory or anti-inflammatory activity [38]. Polarized macrophages are currently classified as either M1 or M2, referring to either classical or alternative activation, respectively [40, 43]. Proinflammatory M1 macrophages arise from exposure to the T-helper (Th)1 cytokines interferon-(IFN)*γ* and tumor necrosis factor (TNF)*α*, in addition to lipopolysaccharide (LPS) or endotoxin [43, 44]. However, polarization of M2 macrophages is more complex than M1 polarization, with three possible subtypes currently defined, each one with diverse physiological roles. Alternatively activated or M2a macrophages are commonly associated with advanced stages of tissue repair and wound healing, arising from exposure to Th2 cytokines such as IL-4 and IL-13. As well as promoting the proliferation of nonmyeloid cells, IL-10 is also known to induce M2c macrophages which have an anti-inflammatory function. Similarly, M2b macrophages also have an anti-inflammatory role and can release large amounts of IL-10. M2b share many features with tumor-associated macrophages [45]. Like M1 macrophages, M2b macrophages also release proinflammatory cytokines, such as IL-1*β* and TNF*α*, but not IL-12.

Proinflammatory macrophages, observed experimentally in the context of muscle repair, are phenotypically similar to classically activated M1 macrophages, and are usually found at early stages after muscle injury, closely followed by macrophages sharing features with the anti-inflammatory M2c phenotype, so-called because of their role in deactivating M1 macrophages [38]. Early on, M1 macrophages phagocytose necrotic muscle debris and participate in the processing and presentation of antigens. In addition to producing high levels of proinflammatory cytokines, M1 macrophages also express inducible nitric oxide synthase (iNOS), which is required to efficiently metabolize L-arginine, a fundamental reaction for producing an abundance of NO for killing intracellular pathogens during infection. Alternatively activated M2a macrophages are more abundant during the final phase of tissue repair [46]. Importantly, M2a macrophages have also been linked to fibrosis in dystrophic *mdx* mouse muscles [39, 47]. M2b-like macrophages have recently been described in regenerating muscle after acute injury [48], suggesting that a wide range of M2 macrophage subtypes might be functional during the muscle repair process.

Despite the emergence of compelling evidence for the presence of different macrophage subtypes in muscle repair, a clear understanding of their specific functions is still lacking. By analogy with the *in vitro* cytokine proinflammatory profile, monocytes entering the muscle at the onset of inflammation resemble M1 polarized macrophages. Indeed, they produce large amounts of the proinflammatory cytokines TNF*α* and IL-1*β* and have an enhanced expression of iNOS. In cell culture models, proinflammatory macrophages have been shown to exert a positive influence on myoblast proliferation while repressing myoblast differentiation [17, 38, 49]. As the process of muscle regeneration advances, they switch their phenotype to resolve inflammation and start to express high levels of IL-10, TGF*β*, and other anti-inflammatory cytokines that dampen the initial cytokine storm. These cytokines have essential roles in promoting proper wound healing, by supporting myogenesis [50], enhancing angiogenesis, and stimulating the transient deposition of the ECM [51]. Similarly, the later wave of anti-inflammatory macrophages stimulates both myoblast differentiation and fusion *in vitro* [17, 38, 49]. The relevance of these inflammatory cells *in vivo* was shown after depletion of blood monocytes exerted negative effects on the regeneration process [38]. Indeed, it is blood monocytes that are the likely source of M1 and M2 macrophages in injured muscle. It is worth noting that although the *in vitro* models of macrophage polarization are useful to establish a theoretical classification, these macrophage populations most likely represent the extremities of a continuum of possible activation states. In addition to problems of classifying polarized macrophages in different tissues and repair models, a recent study has also suggested that there is considerable heterogeneity in the gene expression pattern of different resident macrophage populations in different tissues [52]. Therefore, caution is suggested when classifying wound healing macrophages in general, and muscle infiltrating macrophages in particular, especially when comparing them with *in vitro* polarized macrophages. It is tempting to propose that, rather than belonging to one of these categories, wound healing macrophages could themselves constitute a unique class based on their common characteristics with M1, M2a, M2b, or M2c macrophage subtypes [53]. Thus, to avoid the confusion that can arise from the mixed phenotypes found *in vivo*, some authors propose to classify macrophages regarding their function (i.e., host defense, wound healing, or immune regulation) rather than grouping them on the basis of expression of certain markers [54]. 

## 6. Mechanisms of Macrophage Polarization and Deactivation during Muscle Repair

The mechanisms underlying the transition of macrophage phenotypes during muscle repair are poorly understood. However, certain analogies can be established between *in vitro* macrophage responses to endotoxin and the phenotypic transitions that occur during wound healing. For example, the cAMP response element-binding protein (CREB) plays an important role in generating the anti-inflammatory macrophage phenotype in response to LPS. This response is mediated by the mitogen- and stress-activated kinases 1 and 2 (MSK1 and MSK2), which are, in turn, activated by p38 MAP kinase (MAPK) [55, 56]. In a model of toxic contact eczema induced by phorbol-12-myristate-13-acetate, the CREB-induced expression of IL-10, and dual specificity protein phosphatase 1/MAP kinase phosphatase-1 (DUSP1/MKP-1) inhibited the expression of proinflammatory genes associated with M1 macrophage activation, thus supporting a link between p38/MAPK-1 and CREB in macrophage polarization. An important regulatory function for CREB in macrophage polarization has also been revealed during tissue repair. Indeed, M2, but not M1, macrophage gene expression was impaired by deleting two CREB-binding sites from the C/EBP*β* gene promoter, resulting in abnormal muscle regeneration [57]. Macrophages from the C/EBP*β* promoter mutant mice had a reduced expression of the M2-associated arginase gene after LPS stimulation. It was hypothesized that this may lead to a switch in arginine metabolism from arginase-mediated polyamine synthesis to iNOS-mediated NO production [57]. Importantly, additional studies showed that shifts in macrophage polarization and macrophage competition for arginine metabolism influenced the severity of muscle pathology in *mdx* dystrophic mice [47]. These studies strongly support the idea that CREB might be a pivotal transcription factor in macrophage polarization that functions by promoting M2-associated genes while repressing M1 activation, with CREB transcriptional activity regulated by balance of p38/MSK1/2-MKP-1 activities. 

 Although macrophages sustain proper healing by secreting growth factors and cytokines that support myogenesis and promote transient ECM deposition, dysregulation of the expression of cytokines such as TGF*β* or IL-1*β* can lead to aberrant repair, including fibrosis development, especially in muscle pathologies and conditions characterized by chronic inflammation [21]. Consequently, efficient muscle repair requires resolution of inflammation, and in particular, deactivation of macrophages, at advanced stages of tissue recovery [58]. 

The regulatory mechanisms controlling cytokine gene silencing and macrophage deactivation remain largely undeciphered. One recent study investigated the AKT activation status in macrophages of wild-type and MKP-1-deficient mice during the resolution of inflammation after muscle injury. The activity of AKT was higher in MKP-1^−/−^ than in wild-type macrophages in the late stage of muscle repair, correlating with a loss of pro- and anti-inflammatory cytokine gene expression, and this effect could be reverted by pharmacologically inhibiting p38 MAPK activity [49]. Conversely, macrophages from wild-type mice treated with the PI3K/AKT inhibitor wortmannin showed a prolonged activation status presumably by preventing deactivation. Furthermore, levels of the phosphatase PTEN, which functions as a tumor suppressor by negatively regulating the AKT/PKB signaling pathway, were lower in macrophages in the absence of MKP-1 during the later stage of muscle repair. PTEN is also a direct target of miR-21 [59], a miR classically associated with cancer and fibrosis [60, 61], and its expression was previously reported in RAW 264.7 macrophages [62]. Because miR-21 expression was shown to increase in deactivated macrophages in a p38-dependent manner, [49] it is possible that the loss of MKP-1 (through regulation of the miR-21/PTEN/AKT pathway) extends macrophage cell persistence at the site of injury while at the same time provoking their premature deactivation during the tissue repair process. Taken together, these results strongly support a role for MKP-1 in neutralizing p38 MAPK and thereby controlling sequential macrophage activation-deactivation transitions during tissue repair by restraining AKT activation. 

## 7. Additional Immune Cell Types, Such as Lymphocytes, Are Also Implicated in Muscle Repair and Fibrosis

Macrophages are the predominant inflammatory cells in skeletal muscle regeneration, yet other immune cells, in particular T lymphocytes, have also been proposed to influence repair and fibrosis. Like macrophages, T lymphocytes can also differentiate into distinct functional subsets. The two major types are termed Th1 and Th2, which have distinct roles in orchestrating the host response by generating distinct cytokine profiles [44], whereas more newly characterized including Th17 and Treg subtypes have a less defined role in muscle regeneration, although growing evidence suggests they may become important [63]. Cytokines produced in T cells also regulate muscle degeneration and repair. CD4^+^Th1 cells promote cell-mediated immunity and are able to produce cytokines with antifibrotic properties such as IFN*γ*, TNF*α*, IL-12, and IL-2. By contrast, CD4^+^Th2 cells produce IL-4, IL-5, IL-6, and IL-13, which are cytokines whose primary role is to promote humoral immunity in addition to having profibrotic roles. Importantly, Th1 cytokines inhibit the development of Th2 cells, and conversely, Th2 cytokines inhibit the development of Th1 cells. Clearly, alterations or imbalances in these pathways have the potential to skew repair towards anti- or profibrotic pathways, as witnessed by the importance of Th2 cytokines in the development of liver fibrogenesis [46]. Moreover, T-cell-derived cytokines have a clear role in maintaining the polarized state of macrophages *in vivo*, at least in other models of injury and repair such as after parasite infection [64]. However, the contribution of T cells to macrophage polarization in sterile injury models where T cells are less abundant remains to be explored.

Several studies have suggested roles for T lymphocytes in muscle regeneration. For example, knockout mice lacking the proteolytic activity of the serine protease uPA, and its downstream proteolytic cleavage enzyme plasmin, displayed reduced macrophage and T-lymphocyte infiltration of injured muscle and persistent myofiber degeneration [65–67]. Another study in mice deficient for the *Cbl-b* ubiquitin ligase tumor suppressor gene showed increased infiltration of CD8^+^ T cells into injured muscles with a subsequent delay in muscle regeneration [68]. Deficiency of *Cbl-b* also significantly increased production of the chemokine CCL5 (RANTES) from macrophages during muscle regeneration, whereas neutralization of CCL5 improved the defective muscle regeneration in *Cbl-b-*deficient mice. All together, these results suggest that *Cbl-b* is an important regulator of CD8^+^ T-cell infiltration into regenerating muscle, an effect mediated via CCL5 production in macrophages [68]. In another example, athymic BALB/c nude mice, which are T cell deficient, showed significant increase in central nucleation and increased MMP-9 activity in comparison to wild-type BALB/c [21]. 

Lymphocytes have also been implicated in the deficient regeneration and development of fibrosis observed in some degenerative myopathies. Early studies identified the presence of T cells and several other inflammatory cell subtypes in biopsies of human DMD patients and other myopathies [69, 70]. However, T-cell-mediated cytotoxicity appeared to be limited in DMD patients, despite the appearance of major histocompatibility complex I (MHC I) on regenerating fibers [71]. Dystrophic scid/*mdx* mice, which are deficient in functional T and B lymphocytes, develop much less diaphragm fibrosis with age compared with normal *mdx* mice, concomitant with a decrease in activated TGF*β* in skeletal muscle, [72]. In nu/nu/*mdx* mice (immunodeficient nude mice in the *mdx* background), the lack of functional T cells alone was associated with less diaphragm fibrosis at 3 months, supporting the pathogenic role for T cells in *mdx* muscle and revealing this lymphocyte subclass to be an important source of TGF*β* [73]. A specific subpopulation of T cells expressing the Vb8.1/8.2 T-cell receptor (TCR) was recently identified and shown to be enriched in *mdx* muscle. These T cells produce high levels of osteopontin, a cytokine that promotes immune-cell migration and survival [74]. Intriguingly, osteopontin levels are increased in patients with DMD and in *mdx* mice after disease onset. Importantly, loss of osteopontin in *mdx* double-mutant mice diminishes the infiltration of natural killer T-cell-(NKT-)like cells, which express both T and NK cell markers and neutrophils. These mice also show reduced levels of TGF*β*. These results correlate well with improvements in muscle strength and reduced fibrosis in the diaphragm and heart [74]. Thymectomy at one month of age induces near complete postnatal depletion of circulating T cells in *mdx* mice. When this was followed by anti-CD4 and/or anti-CD8 antibody treatment, it failed to improve diaphragm fibrosis at six months of age [72, 75, 76]. Finally, a recent study investigated the role of lymphocytes in muscle dysferlinopathy using Scid/A/J transgenic mice and showed that the absence of T and B lymphocytes resulted in an improvement of muscle regeneration [77]. 

 Several studies have also shown that mast cells may play a role in normal skeletal muscle repair. Mast cells were shown to accumulate in injured muscles from around 8 hours after saline injection of the gastrocnemius muscle, most of which were recruited from the circulation as very few mast cells are resident in the tissue [78]. Interestingly, mast cells have been linked to development of fibrosis and were shown to be persistently present in *mdx* muscle tissue close to major vessels [78, 79]. Several studies in *mdx* mice and human clinical trials have explored the use of mast cell stabilizers like Oxatomide (Tinset) or Cromolyn (sodium cromoglycate) on the ability to improve muscle repair [80, 81]. Although mast cells are known to release many proinflammatory cytokines such as TNF*α* and interleukins such as IL-1 and IL-6, consistent with their early appearance in muscle after acute damage, in addition to histamine and proteases such as chymase, their role in macrophage polarization is unknown and their overall contribution to efficient repair requires further investigation.

 The above data serves to demonstrate the complexity of the mechanisms that regulate inflammation, muscle repair, and fibrosis development. It is still not clear whether distinct types of Th responses and macrophage subtypes operate in dystrophic muscle, and how they mediate their interactions. Thus, despite our increasing understanding of these immune cells, the implication of the presence of lymphocytes and their subtypes in muscle repair clearly requires further study.

## 8. Concluding Remarks and Future Directions

Numerous recent studies have expanded our knowledge of the function of macrophages, which extends far beyond their role in host defense against bacteria or parasites. The progress in this field has led to the discovery of an increasing number of macrophage activation states, rendering their classification more difficult. If the *in vitro* studies on macrophages activation and their subsequent classification in M1 and M2 macrophages have been useful to mirror the Th1 and Th2 polarization of T cells, the M2 designation has expanded to include all of the non-M1 macrophages. Consequently, a growing number of immunologists now classify them in the extended family of M2-like macrophages. However, the plasticity of these cells makes it difficult to assign specific markers to each population, especially since phenotypic changes are temporally dynamic and depend on changes in the microenvironment and on cell intrinsic mechanisms, like in endotoxin tolerance, which represents a switch from a proinflammatory M1 phenotype to an M2-like anti-inflammatory phenotype. The discovery of new markers, together with progress in flow cytometry techniques, will probably increase even more the complexity of classifying macrophages, rendering it essential to rethink the way we create categories of macrophages and forcing us to focus on their function in order to define these different populations more precisely. Finally, in addition to more precisely defining and evaluating macrophage functions in tissue repair, future research should also focus on identifying in greater detail the function of alternative immune cell types, such as lymphocytes, in the correct resolution of tissue injury or, conversely, in facilitating fibrosis development.
